# Overlap at the molecular and immunohistochemical levels between angioimmunoblastic T-cell lymphoma and a subgroup of peripheral T-cell lymphomas without specific morphological features

**DOI:** 10.18632/oncotarget.24592

**Published:** 2018-03-01

**Authors:** Rebeca Manso, Julia González-Rincón, Manuel Rodríguez-Justo, Giovanna Roncador, Sagrario Gómez, Margarita Sánchez-Beato, Miguel A. Piris, Socorro M. Rodríguez-Pinilla

**Affiliations:** ^1^ Pathology Department, Fundación Jiménez Díaz, UAM, Madrid, Spain; ^2^ Instituto Investigación Sanitaria Puerta de Hierro-Segovia de Arana (IDIPHIM), Madrid, Spain; ^3^ UCL Cancer Institute, Department of Research Pathology, London, UK; ^4^ Monoclonal Antibodies Unit, Biotechnology Programme, Spanish National Cancer Research Centre (CNIO), Madrid, Spain; ^5^ Centro de Investigación Biomédica en Red Cáncer (CIBERONC), Madrid, Spain

**Keywords:** AITL, PTCL, T_FH_-phenotype, IHQ, NGS

## Abstract

The overlap of morphology and immunophenotype between angioimmunoblastic T-cell lymphoma (AITL) and other nodal peripheral T-cell lymphomas (n-PTCLs) is a matter of current interest whose clinical relevance and pathogenic background have not been fully established. We studied a series of 98 n-PTCL samples (comprising 57 AITL and 41 PTCL-NOS) with five T_FH_ antibodies (CD10, BCL-6, PD-1, CXCL13, ICOS), looked for mutations in five of the genes most frequently mutated in AITL (*TET2*, *DNMT3A, IDH2, RHOA* and *PLCG1*) using the Next-Generation-Sequencing Ion Torrent platform, and measured the correlations of these characteristics with morphology and clinical features. The percentage of mutations in the *RHOA* and *TET2* genes was similar (23.5% of cases). *PLCG1* was mutated in 14.3%, *IDH2* in 11.2% and *DNMT3A* in 7.1% of cases, respectively. In the complete series, mutations in *RHOA* gene were associated with the presence of mutations in *IDH2, TET2* and *DNMT3A* (*p <* 0.001, *p =* 0.043, and *p =* 0.029, respectively). Fourteen cases featured *RHOA* mutations without *TET2* mutations. A close relationship was found between the presence of these mutations and a T_FH_-phenotype in AITL and PTCL-NOS patients. Interestingly, BCL-6 expression was the only T_FH_ marker differentially expressed between AITL and PTCL-NOS cases. There were many fewer mutated cases than there were cases with a T_FH_ phenotype. Overall, these data suggest alternative ways by which neoplastic T-cells overexpress these proteins. On the other hand, no clinical or survival differences were found between any of the recognized subgroups of patients with respect to their immunohistochemistry or mutational profile.

## INTRODUCTION

Peripheral T-cell lymphomas (PTCLs) are a heterogeneous group of non-Hodgkin lymphomas (NHLs), characterized by their striking clinical and biological heterogeneity and non-specific therapeutic regimens. In our field, nodal PTCLs (n-PTCLs) are the most frequently diagnosed, and these may be classified into three subgroups: angioimmunoblastic T-cell lymphoma (AITL), peripheral T-cell lymphoma without specific features (PTCL-NOS), and ALK-positive and ALK-negative anaplastic large T-cell lymphoma (ALCL). Diagnostic criteria to distinguish between AITL and PTCL-NOS are mainly based on morphological examination, although an intermediate category has been recognized (PTCL-NOS with T_FH_ markers) [1]. Gene expression array studies indicated that AITL samples were significantly enriched in genes up-regulated in T_FH_ cells [2–4]. Furthermore, the molecular signature of CD30-negative PTCL-NOS partially overlapped with that of T_FH_ cells, although the correlation was not as strong as that with AITLs [2], suggesting that the AITL spectrum may be wider than suspected, as a subset of CD30(-) PTCL-NOs may derive from, or be related to, AITL [2]. We and other researchers have shown that there is also overlap between AITL and some PTCL-NOSs at the morphological and immunohistochemical profile levels [2–4]. Recently, it has been shown that some of these PTCL cases also share most of the molecular background described in AITL samples [2, 4–7]. Tumors that share the T_FH_ immunophenotype (more than two T_FH_ markers) are recognized as the PTCL with T_FH_ phenotype and occupy a distinct provisional category in the new WHO classification [1]. Nevertheless, the criteria for identifying these patients and their clinical characteristics are not currently fully defined. We have studied a series of 98 n-PTCLs samples (comprising 57 AITL and 41 PTCL-NOS cases) with five T_FH_ antibodies (CD10, BCL-6, PD-1, CXCL13, ICOS), and looked for mutations in five of the genes most frequently mutated in AITL (*TET2*, *DNMT3A*, *IDH2*, *RHOA*, *PLCG1*) using the NGS Ion Torrent platform. We have examined the associations of these characteristics with morphological and clinical features. We found a tendency for mutated genes and T_FH_ markers to cluster independently and with each other. Although more frequently found in the AITL patients, a cluster of cases carrying mutated genes and T_FH_ markers were also found in the PTCL-NOS subgroup of tumors.

## RESULTS

### Immunohistochemical study

**Figure 1:**
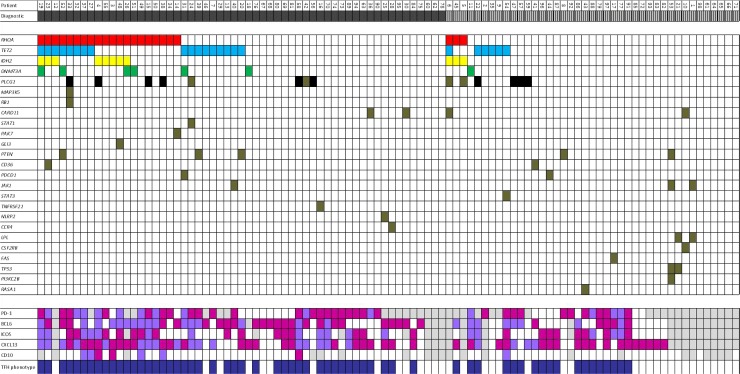
Representative association between mutations of selected genes and T_FH_ markers in n-PTCL according to morphology Dark grey: AITL; Light grey with stripes: PTCL-NOS; Dark blue: T_FH_-phenotype; White: wild-type/no expression; Purple: T_FH_ > 10%; Fuchsia: T_FH_ > 50%; Light grey: no data.

**Figure 2:**
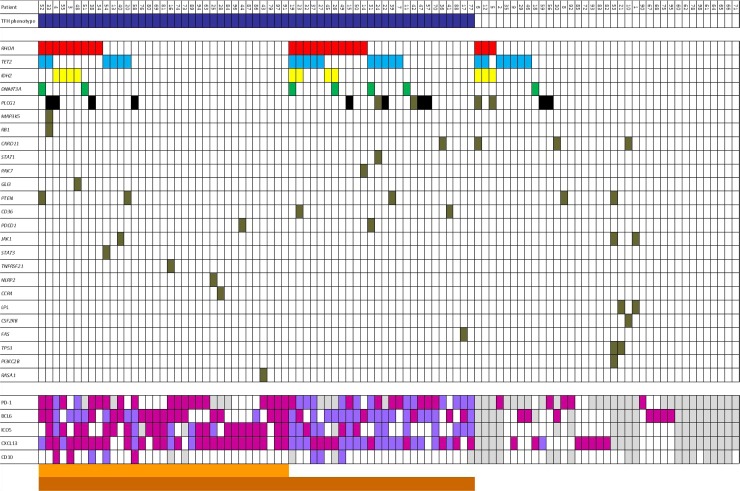
Representative association between mutations of selected genes and cases in n-PTCL according to presence/absence of T_FH_-phenotype Dark blue: T_FH_-phenotype; White: wild-type/no expression; Purple: T_FH_ > 10%; Fuchsia: T_FH_ > 50%; Dark orange: 0–1 markers to 50%; Orange: 2–5 markers to 50%; Light grey: no data.

According to the revised version of the WHO classification of lymphoid tumors, the T_FH_ phenotype is defined as the expression of two or more (ideally, three) T_FH_-related proteins. However, neither the specific markers nor the intensity and percentage of positive cells have been defined precisely. Accordingly, 89.7% (61/68) of our cases had a T_FH_ phenotype when only 10% of tumoral cells (defined as atypical T-cells) expressed at least two of the markers studied (T_FH_-1 group). When the cut-off value for the presence of positive cells for each marker was set at 50% for each marker 51.5% (35/68) of cases were of the T_FH_ phenotype (T_FH_-2 group). The presence of T_FH_ markers occurred at a higher frequency in AITL than PTCL-NOS cases (Supplementary Table 1) (*p* = 0.068 with two or more markers; *p* = 0.059 with three or more markers). On the basis of clusters 1 and 2 (Figures 1 and 2) a 10% cut-off value was chosen for use in the subsequent study.

The most frequently found positive T_FH_ marker was CXCL13, which was positive in 72.94% (62/85) of cases, followed by PD-1 (71.42%, 45/63 cases), BCL-6 (64.63%, 53/82 cases), ICOS (50.63%, 40/79 cases) and CD10 (10.39%, 8/77).

The percentage of positive markers in the AITL group was 77.5% (31/40) for PD-1, 76.9% (40/52) for BCL-6, 73.6% (39/53) for CXCL13, 56.3% (27/48) for ICOS and 14.9% (7/47) for CD10. In analyzing the PTCL-NOS group, the highest frequency of staining was seen in CXCL13 (71.9%, 23/32), followed by PD-1 (60.9%; 14/23), BCL-6 (43.3%; 13/30), ICOS (41.9%, 13/31) and CD10 (3.3%; 1/30). BCL-6 was the only marker differentially expressed between the two subgroups, whereby there was a significantly higher level of expression in the AITL subgroup (*p* = 0.002) (Supplementary Table 2). Double immunohistochemistry for BCL-6/PD-1 was performed on TMA sections. Thirty-two of 79 valuable cases (40.5%) expressed both markers, being more frequent in the AITL subgroup of tumors (*p* = 0.038) (Supplementary Table 2 and Supplementary Figure 1).

Four AITL cases (7%) showed no T_FH_ markers (Supplementary Table 1).

### Mutational study

An equal percentage of cases (23.5%) exhibited mutations in the *RHOA* and *TET2* genes. *PLCG1*, *IDH2* and *DNMT3A* were mutated in 14.3% (14/98), 11.2% (11/98) and 7.1% (7/98) of the cases (Supplementary Table 3).

The percentage of mutations varied between the tumors subgroups. In AITL cases, *RHOA, TET2, IDH2, PLCG1* and *DNMT3A* were mutated in 35.1% (20/57), 29.8% (17/57), 14.03% (8/57), 14.03% (8/57) and 8.8% (5/57) of the cases, respectively (Figure 2).

Conversely, in PTCL-NOS, *TET2, PLCG1, RHOA, IDH2* and *DNMT3A* were mutated in 14.6% (6/41), 14.6% (6/41), 7.3% (3/41), 7.3% (3/41), and 4.9% (2/41) of the cases, respectively (Figure 2).

Only the expression of mutations in the *RHOA* gene differed between AITL and PTCL-NOS tumors (*p* = 0.001) (Supplementary Table 4).

The G17V change was the only mutation found in the *RHOA* gene, the alteration occurring in the GTP-binding domain of *RHOA* predicted to have a damaging function (Supplementary Figure 2). *TET2* was the only gene in which two simultaneous mutations were found in two independent cases each, both of them being AITL cases (cases 31 and 39). Most of these gene alterations were missense mutations (52% of cases), mutations leading to premature stop codons (52% of cases) or alterations in splice sites (8.7%). The same TET2-L1340R mutation was found in two cases. This alteration is predicted to have a damaging function and has also been described in at least two previous independent studies [8, 9]. The profile of mutations in the *DNMT3A* gene was similar, with 71.4% missense mutations, 14.2% mutations leading to premature stop codons and 14.2% of alterations in splice sites. Again, only two (R736C and V690D) of the seven mutations (28.6%) found had been previously described [8, 10].

Mutations in the *IDH2* gene were all missense mutations affecting the same codon, although they give rise to different substitutions (four R172S, four R172G and three R172K). All these mutations have been predicted to have a damaging function.

We had previously used qPCR for the *PLCG1* gene analysis to identify 10/98 cases (10.2%) in this series (represented in black in the cluster) with the PLCG1-S345F mutation (6 AITL and 4 PTCL-NOS) [11]. We have identified four other mutations, three of them missense mutations (Y509H; G1248A and E589V) and one of them an alteration in the 3`UTR region. None of them has been previously described.

In the whole series, mutations in the *RHOA* gene were related to the presence of mutations in the *IDH2, TET2* and *DNMT3A* genes (*p <* 0.005; *p <* 0.043; and *p <* 0.029, respectively). No associations were found between any of the other genes. One of the cases with two double *TET2* mutations also had a *DNMT3A* mutation, while none of these cases showed alterations in the *RHOA* gene.

The variant allele frequency was higher for *TET2* mutations (median, 25.22%; range, 5–64 alleles per case) than for *RHOA* mutations (median, 12.65%; range 5–34 alleles per case). There were 14 cases with *RHOA* mutations without *TET2* mutations. Six of these cases had *IDH2* mutations, one had a *DNMT3A* gene mutation and another had a mutation in both the *IDH2* and *DNMT3A* genes. Only two of the cases with both the *RHOA* and *IDH2* mutations showed a greater than 10% variant allele frequency for the two genes (cases 45 and 48). Six further cases showed no other change in any of these epigenetic-related genes, four of which had a variant allele frequency greater than 10% (Supplementary Table 3).

In the AITL subgroup of tumors, the relationship between mutations in the *RHOA* gene with mutations in both *IDH2* and *DNMT3A* (*p <* 0.005 and *p* = 0.0028) was maintained, while the relationship with mutations in the *TET2* gene was lost. By contrast, in the PTCL-NOS subgroup of tumors, mutations in the *RHOA* gene were associated with *IDH2* (*p <* 0.005) and *PLCG1* (*p* = 0.008) gene mutations. A strong positive relationship was also found between *IDH2* and *PLCG1* (*p* = 0.008). Interestingly, 42.1% (24/57) of AITL cases did not show any of these studied mutations (Figure 1).

No correlations were found between any individual mutation or mutational combination with any of the analyzed clinical parameters (Supplementary Tables 5–9).

### Correlations between the presence of T_FH_ markers and mutations in selected genes

In the whole series, the expression of PD-1 was significantly positively correlated with the presence of mutations in the *TET2* (*p* = 0.044) and *PLCG1* (*p* = 0.034) genes. CXCL13 was positively correlated with the presence of *RHOA* mutations (*p* = 0.002). CD10 was correlated with the presence of mutations in the *PLCG1* (*p* = 0.05) and *RHOA* (*p <* 0.001) genes (Supplementary Table 10). In the AITL subgroup of tumors the correlations between CXCL13 expression and *RHOA* mutations (*p* = 0.001), and between CD10 expression and *PLCG1* (*p* = 0.027) and *RHOA* (*p* = 0.003) gene mutations were maintained. Moreover, a positive association was found between the expression of CD10 and the occurrence of mutations in the *TET2* gene (*p* = 0.010).

A trend between the T_FH_-1 group and *RHOA* gene mutations was found (*p* = 0.068) while no correlations were found regarding T_FH_-2 group. Moreover, the presence of three or more T_FH_ markers was related to the presence of *RHOA* gene mutations (*p* = 0.004).

The presence of double immunohistochemical expression of BCL-6/PD-1 was positively correlated with the presence of mutations in the *RHOA* (*p* = 0.004), *IDH2* (*p* = 0.036) and *PLCG1* (*p* = 0.009) genes (Supplementary Table 10).

## DISCUSSION

In the present series, and in accordance with other published reports [8, 12–14], the presence of T_FH_ markers was broadly associated with AITL morphology. All T_FH_ markers tended to cluster together, and mutations in the five genes studied also clustered together, occurring at a higher frequency in the AITL subgroup of tumors. However, a subgroup of PTCL-NOS showing mutations in these genes as well as a T_FH_ phenotype could also be identified. Additionally, some AITL cases lack T_FH_ markers or the distinctive mutational events. Thus, a grey area can be identified between a cluster of AITL cases with typical morphology, multiple T_FH_ markers and presence of mutated genes with PTCL-NOS without any T_FH_ marker or mutated gene. We have looked for clinical correlations that could help to identify thresholds or case clusters, but failed to identify any, although this could be due to the relatively small number of cases considered here.

In the present series, CXCL13, PD-1, BCL-6, ICOS and CD10 were the most frequently positive markers, occurring in 72.94%, 71.42%, 64.63%, 50.63% and 10.36% of cases, respectively. ICOS expression in this subgroup of patients has not been thoroughly studied. Most studies indicate that CXCL13 is the most frequently expressed gene in AITL and PTCL-NOS samples. Major differences arise from the low frequency of CD10-positive cases found in this study, which could be due to the antibody used or the threshold applied. The level of expression of T_FH_ markers in the PTCL-NOS subgroup of tumors is slightly higher than previously described, ranging from 11% to 61% across different series [4, 15–22]. This discrepancy could be due to an enrichment of PTCL-NOS cases with AITL-like morphology in the present series. 27.3% of the n-PTCL cases in this series exhibited four of the five markers analyzed. Only 7% of AITL cases showed none of the markers, while 43.9% of the PTCL-NOS showed at least two of them.

It is of particular note that BCL-6 was the main marker differentially expressed between AITL and PTL-NOS cases. This relationship was maintained when double PD1/BCL-6 expressers were analyzed (*p* = 0.038). Miyoshi *et al.* [18] reported a relationship between the expression of BCL-6 and AITL morphology. BCL-6 has been described as the master regulator of T_FH_-cells, and can regulate and be regulated by the presence of other T_FH_ cell markers [23]. Many T_FH_ cell markers, such as CXCL13, PD-1 and CD10, can be seen in other T-cell lymphomas such as mycosis fungoides, Sézary syndrome, primary cutaneous T-cell lymphoma with T_FH_-phenotype, or in clonal proliferations of small- to medium-sized CD4-positive T lymphocytes, but BCL-6 is rarely expressed [20, 24]. It is not known whether the presence of BCL-6 is related to the difference in morphology between AITL and T_FH_-PTCL-NOS, or if it has a role in a difference in the etiopathogenesis of these two tumor subgroups.

The percentage of mutated cases in the present series was lower than reported in previous studies (Supplementary Table 11). Variability of tumor-cell content, sequence coverage and efficacy of variant calling probably contributed most to the discrepancy with other reports, most of which found the highest mutation rate to be in the *TET2* gene, followed by *RHOA*. Mutations of *TET2*, *IDH2* and *DNMT3A* usually coexist in AITL patients, unlike the case of myeloid neoplasm, in which *TET2* and *IDH2* mutations appear to be mutually exclusive. Furthermore, the variant allele frequency is higher for *TET2* and *DNMT3A* mutations than for *RHOA* mutations in AITL cases [9]. *TET2* and *DNMT3A* mutations are thought to occur at an early stage of hematopoietic cell differentiation since they are also found in non-malignant hematopoietic cells, non-transformed CD20-positive immunoblasts in AITL patients, as well as in normal elderly individuals. Based on these observations, a multistage developmental pathway for AITL has been suggested, in which *TET2* and *DNMT3A* are both early events related to enhanced self-renewal, while mutations in the *RHOA* gene, among others, are secondary events associated with the malignant transformation of lineage commitment [8–10, 25–28]. However, we found no relationship between *TET2* and *DNMT3A* mutations, although one of the cases with mutations in both genes had two different mutations in the *TET2* gene, in accordance with previous reports. Although the variant allele frequencies of the *TET2* and *DNMT3A* genes were higher than that of the *RHOA* gene, we found six *RHOA* gene-mutated cases that had no other mutations in *TET2*, *DNMT3A* or *IDH2*. In the present series, all *IDH2*-mutated cases also had *RHOA* gene mutations that were present in the AITL and PTCL-NOS patients. Although most authors suggest a close correlation between mutations in *IDH2* and AITL morphology, only one study has yielded results that concur with ours [14].

Only mutations in the *RHOA* gene were differentially expressed between AITL and PTCL-NOS tumors (*p* = 0.001). AITL is pathologically characterized by marked proliferation of endothelial venules, expanded follicular dendritic cell (FDC) meshworks around the venules, diffuse polymorphic infiltrates, and the expansion of EBV-positive or EBV-negative B-immunoblasts [29]. Three histological patterns are recognized, of which the most common is absent follicles, although cases with depleted or hyperplastic follicles have also been described [30]. Our findings concur with those of two previous studies showing the association between the presence of the RHOA-G17V mutation and a classic AITL morphology with expanded dendritic meshwork and T_FH_ phenotype [31, 32].

We and other researchers have described a relatively high percentage of AITL patients without mutations in any of the genes here mentioned. *A priori*, these results suggest that alternative pathogenic events play a role in the development of AITL, at least in a subgroup of these patients. So, alternative ways of changing the same or different genes in these pathways are probably responsible for the development of these non-mutated patients.

*TET2* mutation is the only mutated gene known to be correlated with aggressive clinical features [22]. However, our data do not support this or any other association.

A close relationship was found between the presence of these mutations and a T_FH_-phenotype in both AITL and T_FH_-phenotype PTCL-NOS patients, suggesting the existence of a core of AITL cases carrying both T_FH_ phenotype and mutated genes. Nevertheless, the frequency of mutated cases was much lower than that of cases with a T_FH_ phenotype [23, 33]. No clinical or survival differences were found regarding these subgroups. Combinations of two, three and four T_FH_ markers showed different correlations with mutated genes. *RHOA* gene mutations were associated with most combinations of T_FH_-markers. PD-1/CD10 as well as double-expresser tumors for PD1 and BCL-6 proteins were correlated with the presence of most mutated genes (*RHOA, IDH2* and *PLCG1*). No clinical differences were found in any association between gene mutations and T_FH_-markers. These results make it very difficult to recommend which T_FH_ proteins should be used to identify n-PTCL with T_FH_ phenotype, but it is clear that the use of multiple markers should be accompanied by a standardization of the techniques and interpretation of results.

Except for the *RHOA* and *IDH2* genes, the number and function of mutations in the other genes, especially *TET2* and *DNMT3A*, are highly variable. This means that they are not very useful in daily clinical practice, although they may be of great biological significance, suggesting that AITL pathogenesis could arise in some cases from the clonal expansion of hematopoietic precursor cells. The presence of most of these mutations is not specific to AITL or other T_FH_-PTCL-NOS cases, since they have also been found to be mutated in cutaneous T-cell lymphomas, Sézary syndrome, T-cell prolymphocytic leukemia, adult T-cell leukemia/lymphoma, acute lymphoblastic leukemia, T-cell and NK-cell post-transplant lymphoproliferative disorders, and in diffuse large B-cell lymphomas [34–44]. To the best of our knowledge, the *IDH2* gene has not been found to be mutated in other subgroups of lymphomas, except for exceptional cases of lymphoblastic leukemia [45], making it somehow characteristic of these subgroups of tumors. Unfortunately, they were present in only 11.2% of our cases, compared with the highest reported rate of 33.3% [28]. Nevertheless, the knowledge of the mutational status of n-PTCL samples could be useful as markers for guiding future therapy or patient follow-up [46–48].

In conclusion, AITL differed from T_FH_-PTCL-NOS cases with respect to morphology, BCL-6 expression and *RHOA* mutation rate, although none of these features had clinical implications. In general, the AITL and T_FH_-PTCL-NOS subgroups of tumors share morphological features, immunophenotype, molecular background and clinical behavior. Considering all these features together could justify ascribing all these entities to a single category in the lymphoma classification [1].

## MATERIALS AND METHODS

### Patient samples

The series included 98 formalin-fixed, paraffin-embedded (FFPE) n-PTCL cases (57 AITL and 41 PTCL-NOS). Diagnostic criteria were based on the WHO classification [1]. All samples were reviewed by two pathologists (SMR-P and MAP) to confirm the diagnoses. Patients’ clinical data have been reported in previous publications [11, 12, 49–51]. Patient characteristics are presented in Supplementary Table 12. Samples and clinical data of patients included in the study were provided by several Spanish Biobanks. The project was supervised by the Ethical Committees of the Hospital Universitario Marqués de Valdecilla (Santander) and the Fundación Jiménez Díaz (Madrid).

### Tissue microarray construction

Representative areas from FFPE lymphomas were carefully selected from H&E-stained sections. Three tissue cores of 1 mm diameter were obtained from each specimen. The cores were precisely arrayed into a new paraffin block using a tissue microarray (TMA) workstation (Beecher Instruments, Silver Spring, MD).

### Immunohistochemical studies

TMA sections were stained by the EndVision method with a heat-induced antigen-retrieval step for BCL-6, ICOS, CD10, PD-1 and CXCL13. Reactive tonsil tissue was included as a control. The primary antibodies were omitted to provide negative controls (Supplementary Table 13). Cases were considered to belong to the T_FH_-phenotype subgroup when at least two different markers were positive. Sixty-eight of 98 cases had valuable T_FH_-phenotype data. Two groups were defined on the basis of the percentage of positive cells for each marker (group 1, >10%; group 2, >50%). Double immunohistochemistry for BCL-6/PD-1 was performed on TMA sections. Only 79 of the 98 cases produced valuable data.

### Double immunoenzymatic staining

For paraffin-embedded tissues, an initial automated dewaxing and rehydration step followed by heat-induced (100° C for 20 min) or enzyme-induced (10 to 15 min, Bond Enzyme Pretreatment Kit, Leica Biosystems, Wetzlar, Alemania) antigen retrieval was performed. Heat-induced antigen retrieval was performed using pH 8.8 ethylenediaminetetraacetic acid (EDTA)-based ready-to-use solution (Leica Biosystems). Slides were subsequently incubated with 3% hydrogen peroxide (5 min), optimally diluted primary antibody (15 to 30 min), a postprimary blocking reagent (to prevent nonspecific polymer binding) (8 min), horseradish peroxidase-labeled polymer (8 min), and diaminobenzidine substrate (10 min). All reagents were components of the Bond Polymer Refine detection system (Leica Biosystems). New adhesive labels needed for the second staining procedure were applied to the slides. A second immunophosphatase (AP) procedure was then performed, omitting the dewaxing, rehydration and epitope retrieval steps. The primary antibody was applied for 40 min, followed by incubation with postprimary AP blocking reagent (20 min) and AP-labeled polymer (30 min), both of which are components of the Bond Polymer AP Red detection system (Leica Biosystems). The AP reaction was carried out with the Fast Red substrate included in the Bond Polymer AP Red detection system. Hematoxylin counterstaining was performed.

### DNA extraction

We extracted genomic DNA (DNAg) of tumoral FFPE samples using a QIAamp^®^ DNA FFPE Tissue kit (Qiagen Inc., Valencia, CA, USA) in accordance with the manufacturer’s protocol. DNAs were quantified with Qubit^®^ (Invitrogen, Carlsbad, CA, USA). The quantity and quality of DNAs used in constructing libraries for next-generation sequencing (NGS) were assessed using the KAPA Human Genomic DNA Quantification and QC kit (KAPA Biosystems Inc., Roche) and the 7500 Real Time System (Applied Biosystems, Foster City, CA, USA) in accordance with the manufacturer’s protocol.

### Detection of *PLCG1* mutation by qPCR

We used two previously described methods [11, 41] two detect the S345F mutation of *PLCG1*.

### NGS Custom Panel design

The Ion kit AmpliSeq^™^ Library kit (Life Technologies, Carlsbad, California, USA) panel includes 48 genes being previously found mutated in T-cell lymphomas (Supplementary Table 14) and it was designed using AmpliSeq^™^ Custom Ion panel Designer (Life Technologies) (https://www.ampliseq.com/browse.action).

### Library preparation and sequencing

For targeted sequencing we used 30 ng DNAg and the Ion AmpliSeq^™^ custom panel technology (Life Technologies), following the standard protocol. The quantification of amplifiable library molecules is critical for the efficient use of the Ion Torrent NGS platform; we performed the qPCR using the Library Quantitation kit (Life Technologies) and the StepOne^™^ System (Applied Biosystems, Foster City, CA, USA) in accordance with the manufacturer’s protocol. We then prepared and enriched the template DNA by PCR emulsion performed on Ion Sphere Particles (ISPs). Libraries were sequenced using the Ion Proton^™^ instrument according to the manufacturer’s protocol.

### Bioinformatic analysis

The reads obtained with the sequencer were analyzed using the integrated Torrent Suite system (Life Technologies). The sequences were aligned with the reference genome NCBI Build 37 (UCSC hg19) using TMAP-Ion-Alignment software. The variants were then identified by the Torrent Variant Caller algorithm and the variants were annotated with the Ion Reporter (Life Technologies). Relevant somatic mutations were called and filtered by excluding: (i) synonymous SNVs; (ii) known variants listed in SNP databases (as described above); (iii) variants only present in unidirectional reads; (iv) variants occurring in repetitive genomic regions; (v) variants with coverage of <30X; (vi) variants with fewer than five reads in tumor samples; and (vii) mutations with a Variant Allele Frequency (VAF) of <0.05. The variants were visually checked using Integrated Genome Viewer (IGV v2.3; Broad Institute) to exclude artifacts and sequencing errors. The COSMIC (Catalogue of Somatic Mutations in Cancer) database was checked to identify pathogenetic changes. In addition, the variants were analyzed with two mutational functional prediction programs (SIFT and Polyphen-2).

### Statistical analysis

To assess associations between categorical variables, we used the X^2^ contingency test with Yates’ correction, or Fisher’s exact test, as appropriate. Overall survival (OS) was taken as the period between the date of diagnosis and the date of death from any cause, or of last contact for living patients. Disease-specific OS was calculated as the period from date of diagnosis until death from the tumor. Kaplan–Meier survival analyses were carried out for OS and lymphoma-specific survival, using the log-rank test to examine differences between groups. A multivariate Cox regression model was also derived. Estimates were considered statistically significant for two-tailed values of *p <* 0.05. All analyses were carried out with SPSS v.20.0 (IBM Corp., Armonk, NY, USA) [51].

## SUPPLEMENTARY MATERIALS FIGURES AND TABLES




