# Supplementary material for: Overlap at the molecular and immunohistochemical levels between angioimmunoblastic T-cell lymphoma and a subgroup of peripheral T-cell lymphomas without specific morphological features
Source: Oncotarget. 2018 Mar 1;9(22):16124–33. doi: 10.18632/oncotarget.24592 (PMC5882322; doi:10.18632/oncotarget.24592)
Supplement: Supplementary file 1 [file oncotarget-09-16124-s001.pdf]

Overlap at the molecular and immunohistochemical levels between angioimmunoblastic T-cell lymphoma and a subgroup of peripheral T-cell lymphomas without specific morphological features

SUPPLEMENTARY MATERIALS

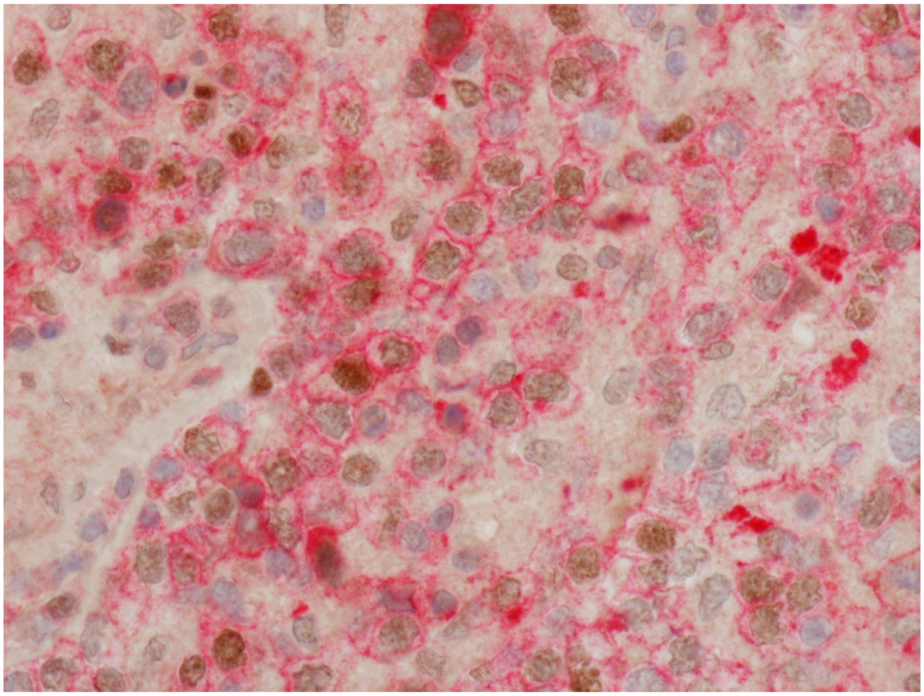

Supplementary Figure 1: An AITL case showing in the majority of neoplastic cells both PD-1 (red) and BCL-6 expression (brown).

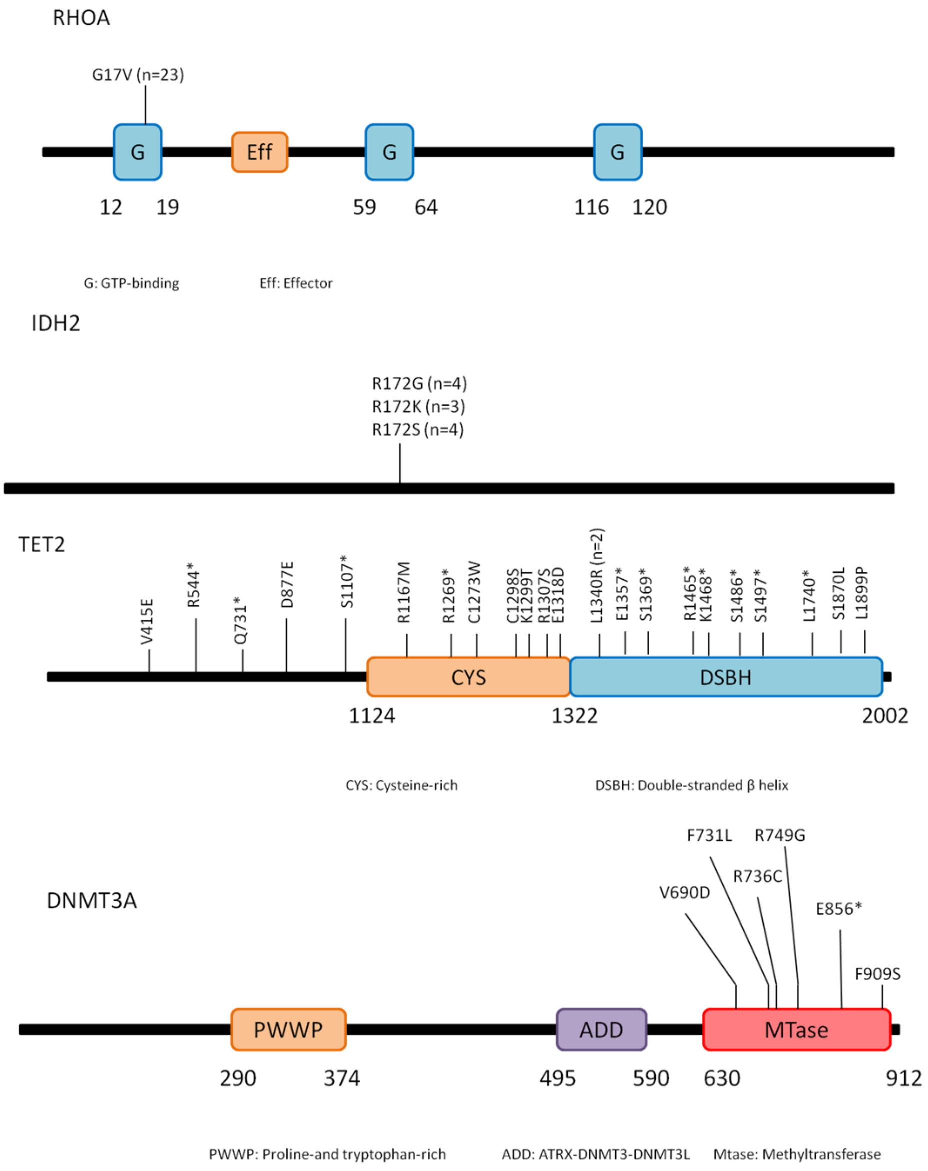

Supplementary Figure 2: Schematic of the structure of the *RHOA, IDH2, TET2* and *DNMT3A* proteins.

Supplementary Table 1: Number of T_{FH} markers in n-PTCL by morphological subtype

TFH numbers	AITL (*n* = 57)	PTCL-NOS (*n* = 41)
0	4 (7%)	13 (31.7%)
1	10 (17.5%)	10 (24.4%)
2	12 (21.1%)	7 (17.1%)
3	17 (29.8%)	6 (14.6%)
4	10 (17.5%)	4 (9.8%)
5	4 (7%)	1 (2.4%)

Supplementary Table 2: Correlation between T_{FH} markers and histological subtypes of n-PTCLs

	AITL	PTCL-NOS	*P*-VALOR
PD-1	31	14	0.159
BCL-6	40	13	0.002
ICOS	27	13	0.214
CXCL13	39	23	0.863
CD10	7	1	0.105
BCL-6/PD-1	25	7	0.038

Supplementary Table 3: Mutational repertoire found in the present series of n-PTCL cases. See Supplementary_Table_3

Supplementary Table 4: Correlations between mutated genes and histological subtypes of n-PTCLs and average coverage of each variant

	Average coverage	AITL (*n* = 57)	PTCL-NOS (*n* = 41)	*p*-valor
RHOA	1149	20	3	0.001
TET2	1154	17	6	0.08
IDH2	1143	8	3	0.299
DNMT3A	1151	6	1	0.46
PLCG1	1178	8	5	0.962

Supplementary Table 5: Univariate analysis of the cohort of 98 patients with n-PTCL by *RHOA* status

	Total cases	WT *RHOA*	MUT *RHOA*	*p*
**DX**	98/98			0.001
AITL		37/57 (64.9%)	20/57 (35.1%)	
PTCL-NOS		38/41 (92.7%)	3/41 (7.3%)	
**Sex**	95/98			0.161
Male		44/60 (73.3%)	16/60 (26.7%)	
Female		30/35 (85.7%)	5/35 (14.3%)	
**Age at diagnosis**	78/98			0.258
<60 years		25/29 (86.2%)	4/29 (13.8%)	
≥60 years		37/49 (75.5%)	12/49 (24.5%)	
**IPI**	86/98			0.725
Low risk		21/25 (84.0%)	4/25 (16.0%)	
Low-intermediate risk		17/24 (70.8%)	7/24 (29.2%)	
High-intermediate risk		14/18 (77.8%)	4/18 (22.2%)	
High risk		14/19 (73.7%)	5/19 (26.3%)	
**PIT**	80/98			0.839
Low risk		8/11 (72.7%)	3/11 (27.3%)	
Low-intermediate risk		26/33 (78.8%)	7/33 (21.2%)	
High-intermediate risk		16/20 (80.0%)	4/20 (20.0%)	
High risk		11/16 (68.8%)	5/16 (31.2%)	
**ECOG**	83/98			0.491
<1		46/59 (78.0%)	13/59 (22.0%)	
≥1		17/24 (70.8%)	7/24 (29.2%)	
**Treatment**	87/98			0.136
CHOP/CHOP-LIKE		54/65 (83.1%)	11/65 (16.9%)	
Others		15/22 (68.2%)	7/22 (31.8%)	
**Response**	77/98			0.032
CR		33/48 (68.8%)	15/48 (31.2%)	
PR		13/13 (100%)	0/13 (0%)	
No response		14/16 (87.5%)	2/16 (12.5%)	
**Recurrence**	75/98			0.098
No		40/48 (83.3%)	8/48 (16.7%)	
Yes		18/27 (66.7%)	9/27 (33.3%)	
**State of the patient**	89/98			0.527
Dead		40/50 (80.0%)	10/50 (20.0%)	
Alive		29/39 (74.4%)	10/39 (25.6%)	

WT: wild type; MUT: mutated; DX: diagnosis; CR: total response; PR: partial response; IPI: International Prognostic Index; PIT: Prognostic Index for PTCL-u.

Supplementary Table 6: Univariate analysis of the cohort of 98 patients with n-PTCL by *TET2* status

	Total cases	WT ***TET2***	MUT ***TET2***	***p***
**DX**	98/98			0.08
AITL		40/57 (70.2%)	17/57 (29.8%)	
PTCL-NOS		35/41 (85.4%)	6/41 (14.6%)	
**Sex**	95/98			0.219
Male		43/60 (71.7%)	17/60 (28.3%)	
Female		29/35 (82.9%)	6/35 (17.1%)	
**Age at diagnosis**	78/98			0.864
<60 years		22/29 (75.9%)	7/29 (24.1%)	
≥60 years		38/49 (77.6%)	11/49 (22.4%)	
**IPI**	86/98			0.196
Low risk		20/25 (80.0%)	5/25 (20.0%)	
Low-intermediate risk		17/24 (70.8%)	7/24 (29.2%)	
High-intermediate risk		10/18 (55.6%)	8/18 (44.4%)	
High risk		16/19 (84.2%)	3/19 (15.8%)	
**PIT**	80/98			0.862
Low risk		9/11 (81.8%)	2/11 (18.2%)	
Low-intermediate risk		24/33 (72.7%)	9/33 (27.3%)	
High-intermediate risk		16/20 (80.0%)	4/20 (20.0%)	
High risk		13/16 (81.3%)	3/16 (18.7%)	
**ECOG**	83/98			0.843
<1		43/59 (72.9%)	16/59 (27.1%)	
≥1		18/24 (75.0%)	6/24 (25.0%)	
**Treatment**	87/98			0.033
CHOP/CHOP-LIKE		53/65 (81.5%)	12/65 (18.5%)	
Others		13/22 (59.1%)	9/22 (40.1%)	
**Response**	77/98			0.686
CR		35/48 (72.9%)	13/48 (27.1%)	
PR		11/13 (84.6%)	2/13 (15.4%)	
No response		12/16 (75.0%)	4/16 (25.0%)	
**Recurrence**	75/98			0.764
No		34/48 (70.8%)	14/48 (29.2%)	
Yes		20/27 (74.1%)	7/27 (25.9%)	
**State of the patient**	89/98			0.969
Dead		37/50 (74.0%)	13/50 (26.0%)	
Alive		29/39 (74.4%)	10/39 (25.6%)	

WT: wild type; MUT: mutated; DX: diagnosis; CR: total response; PR: partial response; IPI: International Prognostic Index; PIT: Prognostic Index for PTCL-u.

Supplementary Table 7: Univariate analysis of the cohort of 98 patients with n-PTCL by *IDH2* status

	Total cases	WT *IDH2*	MUT *IDH2*	*p*
**DX**	98/98			0.299
AITL		49/57 (86.0%)	8/57 (14.0%)	
PTCL-NOS		38/41 (92.7%)	3/41 (7.3%)	
**Sex**	95/98			0.032
Male		50/60 (83.3%)	10/60 (16.7%)	
Female		34/35 (97.1%)	1/35 (2.9%)	
**Age at diagnosis**	78/98			0.839
<60 years		27/29 (93.1%)	2/29 (6.9%)	
≥60 years		45/49 (91.8%)	4/49 (8.2%)	
**IPI**	86/98			0.506
Low risk		24/25 (96.0%)	1/25 (4.0%)	
Low-intermediate risk		20/24 (83.3%)	4/24 (16.7%)	
High-intermediate risk		16/18 (88.9%)	2/18 (11.1%)	
High risk		16/19 (84.2%)	3/19 (15.8%)	
**PIT**	80/98			0.631
Low risk		11/11 (100%)	0/11 (0%)	
Low-intermediate risk		29/33 (87.9%)	4/33 (12.1%)	
High-intermediate risk		17/20 (85.0%)	3/20 (15.0%)	
High risk		14/16 (87.5%)	2/16 (12.5%)	
**ECOG**	83/98			0.117
<1		54/59 (91.5%)	5/59 (8.5%)	
≥1		19/24 (79.2%)	5/24 (20.8%)	
**Treatment**	87/98			0.404
CHOP/CHOP-LIKE		60/65 (92.3%)	5/65 (7.7%)	
Others		19/22 (86.4%)	3/22 (13.6%)	
**Response**	77/98			0.345
CR		42/48 (87.5%)	6/48 (12.5%)	
PR		13/13 (100%)	0/13 (0%)	
No response		15/16 (93.8%)	1/16 (6.3%)	
**Recurrence**	75/98			0.221
No		45/48 (93.8%)	3/48 (6.3%)	
Yes		23/27 (85.2%)	4/27 (14.8%)	
**State of the patient**	89/98			0.274
Dead		46/50 (92.0%)	4/50 (8.0%)	
Alive		33/39 (84.6%)	6/39 (15.4%)	

WT: wild type; MUT: mutated; DX: diagnosis; CR: total response; PR: partial response; IPI: International Prognostic Index; PIT: Prognostic Index for PTCL-u.

Supplementary Table 8: Univariate analysis of the cohort of 98 patients with n-PTCL by *DNMT3A* status

	Total cases	WT *DNMT3A*	MUT *DNMT3A*	*p*
**DX**	98/98			0.46
AITL		52/57 (91.2%)	5/57 (8.8%)	
PTCL-NOS		39/41 (95.1%)	2/41 (4.9%)	
**Sex**	95/98			0.732
Male		56/60 (93.3%)	4/60 (6.7%)	
Female		32/35 (91.4%)	3/35 (8.6%)	
**Age at diagnosis**	78/98			0.189
<60 years		28/29 (96.6%)	1/29 (3.4%)	
≥60 years		43/49 (87.8%)	6/49 (12.2%)	
**IPI**	86/98			0.609
Low risk		23/25 (92.0%)	2/25 (8.0%)	
Low-intermediate risk		22/24 (91.7%)	2/24 (8.3%)	
High-intermediate risk		18/18 (100%)	0/18 (0%)	
High risk		17/19 (89.5%)	2/19 (10.5%)	
**PIT**	80/98			0.31
Low risk		11/11 (100%)	0/11 (0%)	
Low-intermediate risk		30/33 (90.9%)	3/33 (9.1%)	
High-intermediate risk		20/20 (100%)	0/20 (0%)	
High risk		14/16 (87.5%)	2/16 (12.5%)	
**ECOG**	83/98			0.804
<1		55/59 (93.2%)	4/59 (6.8%)	
≥1		22/24 (91.7%)	2/24 (8.3%)	
**Treatment**	87/98			0.436
CHOP/CHOP-LIKE		62/65 (95.4%)	3/65 (4.6%)	
Others		20/22 (90.9%)	2/22 (9.1%)	
**Response**	77/98			0.563
CR		45/48 (93.8%)	3/48 (6.2%)	
PR		12/13 (92.3%)	1/13 (7.7%)	
No response		16/16 (100%)	0/16 (0%)	
**Recurrence**	75/98			0.638
No		45/48 (93.8%)	3/48 (6.2%)	
Yes		26/27 (96.3%)	1/27 (3.7%)	
**State of the patient**	89/98			0.592
Dead		46/50 (92.0%)	4/50 (8.0%)	
Alive		37/39 (94.9%)	2/39 (5.1%)	

WT: wild type; MUT: mutated; DX: diagnosis; CR: total response; PR: partial response; IPI: International Prognostic Index; PIT: Prognostic Index for PTCL-u.

Supplementary Table 9: Univariate analysis of the cohort of 98 patients with n-PTCL by *PLCG1* status

	Total cases	WT *PLCG1*	MUT *PLCG1*	*p*
**DX**	97/98			0.962
AITL		48/56 (85.7%)	5/56 (14.3%)	
PTCL-NOS		35/41 (85.4%)	6/41 (14.6%)	
**Sex**	94/98			0.521
Male		50/60 (83.3%)	10/60 (16.7%)	
Female		30/34 (88.2%)	4/34 (11.8%)	
**Age at diagnosis**	77/98			0.212
<60 years		23/29 (79.3%)	6/29 (20.7%)	
≥60 years		43/48 (89.6%)	5/48 (10.4%)	
**IPI**	85/98			0.592
Low risk		19/25 (76.0%)	6/25 (24.0%)	
Low-intermediate risk		19/23 (82.6%)	4/23 (17.4%)	
High-intermediate risk		16/18 (88.9%)	2/18 (11.1%)	
High risk		17/19 (89.5%)	2/19 (10.5%)	
**PIT**	79/98			0.475
Low risk		9/11 (81.8%)	2/11 (18.2%)	
Low-intermediate risk		29/32 (90.6%)	3/32 (9.4%)	
High-intermediate risk		15/20 (75.0%)	5/20 (25.0%)	
High risk		14/16 (87.5%)	2/16 (12.5%)	
**ECOG**	82/98			0.95
<1		48/58 (82.8%)	10/58 (17.2%)	
≥1		20/24 (83.3%)	4/24 (16.7%)	
**Treatment**	86/98			0.079
CHOP/CHOP-LIKE		57/65 (87.7%)	8/65 (12.3%)	
Others		15/21 (71.4%)	6/21 (28.6%)	
**Response**	76/98			0.409
CR		40/48 (83.3%)	8/48 (16.7%)	
PR		12/13 (92.3%)	1/13 (7.7%)	
No response		11/15 (73.3%)	4/15 (26.7%)	
**Recurrence**	74/98			0.425
No		40/47 (85.1%)	7/47 (14.9%)	
Yes		21/27 (77.8%)	6/27 (22.2%)	
**State of the patient**	88/98			0.904
Dead		41/49 (83.7%)	8/49 (16.3%)	
Alive		33/39 (84.6%)	6/39 (15.4%)	

WT: wild type; MUT: mutated; DX: diagnosis; CR: total response; PR: partial response; IPI: International Prognostic Index; PIT: Prognostic Index for PTCL-u.

Supplementary Table 10: Correlation between pairwise-associated T_{FH} markers and mutated genes in n-PTCLs

	n	*RHOA*	*P*-VALUE	*IDH2*	*P*-VALUE	*TET2*	*P*-VALUE	*DNMT3A*	*P*-VALUE	*PLCG1*	*P*-VALUE
CD10	77	6	<0.001	2	0.055	3	0.374	0	0.431	3	0.05
BCL-6	82	16	0.098	7	0.154	15	0.265	6	0.06	10	0.134
ICOS	79	13	0.075	5	0.249	11	0.468	3	0.974	6	1
PD-1	85	14	0.099	5	0.497	13	0.044	4	0.658	10	0.034
CXCL13	63	20	0.002	8	0.07	15	0.813	7	0.093	10	0.369
BCL-6/PD-1	79	13	0.004	6	0.036	10	0.217	4	0.174	9	0.009

Supplementary Table 11: Mutation rate of selected studied genes involved in n-PTCLs

	Reference	%					Total nº cases
		TET2	*RHOA*	*DNMT3A*	*IDH2*	*PLCG1*	
**AITL**	Cairns *et al.* (2012)	–	–	–	20	–	79
	Lemmonier *et al.* (2012)	47	–	–	–	–	86
	Palomero *et al.* (2014)	47	67	–	–	–	35
	Sakata-Yanagimoto *et al.* (2014)	82.6	70.8	26	30.4	–	72
	Odejide *et al.* (2014)	76	–	33	20	–	85
	Yoo *et al.* (2014)	–	53.3	–	–	2.2	45
	Manso *et al.* (2014)	–	34.7	v	–	–	72
	Manso *et al.* (2015)	–	–	–	–	11.7	60
	Vallois *et al.* (2016)	37.5	62.5	23.6	29.2	11.1	72
	Nagao *et al.* (2016)	–	56	–	–	–	18
	Ondrejka *et al.* (2016)	–	63	–	–	–	27
	Dobay *et al.* (2017)	28.7	40.4	17	21.3	–	94
	Nguyen *et al.* (2017)	75	68.8	22.9	27.1	–	48
	Wang *et al.* (2017)	100	33.3	33.3	33.3	22.2	9
**PTCL-NOS**	Cairns *et al.* (2012)	–	–	–	0	–	43
	Lemmonier *et al.* (2012)	38	–	–	–	–	58
	Palomero *et al.* (2014)	38	18	–	–	–	44
	Sakata-Yanagimoto *et al.* (2014)	48.5	17.2	27.3	0	–	87
	Manso *et al.* (2014)	–	14.6	–	–	–	48
	Manso *et al.* (2015)	–	–	–	–	14.6	41
	Vallois *et al.* (2016)	46.2	46.2	7.7	0	30.8	13
	Ondrejka *et al.* (2016)	–	0	–	–	–	10
	Dobay *et al.* (2017)	16.7	22.2	2.8	2.8	–	36
	Nguyen *et al.* (2017)	61.5	20.5	30.8	0	–	39

Supplementary Table 12: Clinical parameters of the series of 98 patients with n-PTCL

Clinical parameters		
	**No.**	**%**
**Diagnosis**		
AITL	57	58.2
PTCL-NOS	41	41.8
**Sex**		
Male	60	63.2
Female	35	36.8
**Age at diagnosis**		
<60 years	29	37.2
≥60 years	49	62.8
**IPI**		
Low risk	25	29
Low-intermediate risk	24	27.9
High-intermediate risk	18	21
High risk	19	22.1
**PIT**		
Low risk	11	13.7
Low-intermediate risk	33	41.3
High-intermediate risk	20	25
High risk	16	20
**ECOG**		
<1	59	71.1
≥1	24	28.9
**Treatment**		
CHOP/CHOP-LIKE	65	74.7
Others	22	25.3
**Response**		
CR	48	62.3
PR	13	16.9
No response	16	20.8
**Recurrence**		
No	48	64
Yes	27	36
**State of the patient**		
Dead	50	56.2
Alive	39	43.8

Supplementary Table 13: Panel of antibodies used in this series

Antibody	*Clone*	*Source*	*Cut-off value for positivity*	*number of valuables*
BCL-6 FLEX	Mouse monoclonal (PG-B6p)	DAKO	>10%	82
PD-1	Mouse monoclonal (NAT)	CNIO	>10%	63
ICOS	Rabbit polyclonal (FL-199)	Santa Cruz Biotechnology	>10%	79
CXCL13	Goat polyclonal (AF801)	R&D Systems	>10%	85
CD10 FLEX	Mouse monoclonal (56C6)	DAKO	>10%	77

Supplementary Table 14: Panel of genes sequenced using the NGS Ion Torrent platform in this series of patients

AKT2	*IDH2**	*PAK7*	*RHOA**
BANK1	*IL6ST*	*PASK*	*SOCS5*
BCOR	*ITGAM*	*PDCD1*	*SPI1*
CARD11	*JAK1**	*PIK3C2B*	*STAT1*
CCR4	*JAK3**	*PIK3CA*	*STAT3**
CD36	*KRAS*	*PIK3R1*	*STAT5A*
CD79A	*LPL**	*PLCG1*	*TBK1*
CSF2RB	*MAP3K14*	*PTEN*	*TET2*
CYLD	*MAP3K5*	*RASA1*	*TNFRSF21*
*DNMT3A**	*MAPK14*	*RB1*	*TP53**
FAS	*NLRP2*	*RC3H1*	*TRAF3*
GLI3	*NRAS*	*RELB*	*TRAF6*

*Specific regions.
